# A Multi-Center Cohort Study on Characteristics of Pain, Its Impact and Pharmacotherapeutic Management in Patients with ALS

**DOI:** 10.3390/jcm10194552

**Published:** 2021-09-30

**Authors:** Susanne Vogt, Ina Schlichte, Stefanie Schreiber, Bernadette Wigand, Grazyna Debska-Vielhaber, Johanna Heitmann, Thomas Meyer, Reinhard Dengler, Susanne Petri, Aiden Haghikia, Stefan Vielhaber

**Affiliations:** 1 Department of Neurology, Otto-von-Guericke University, 39120 Magdeburg, Germany; ina.schlichte@gmx.de (I.S.); stefanie.schreiber@med.ovgu.de (S.S.); bernadette-wigand@gmx.de (B.W.); grazyna.debska-vielhaber@med.ovgu.de (G.D.-V.); johanna.heitmann@med.ovgu.de (J.H.); aiden.haghikia@med.ovgu.de (A.H.); stefan.vielhaber@med.ovgu.de (S.V.); 2 German Center for Neurodegenerative Diseases (DZNE), 39120 Magdeburg, Germany; 3 Center for Behavioral Brain Sciences (CBBS), 39106 Magdeburg, Germany; 4 Department of Neurology, Center for ALS and other Motor Neuron Disorders, Charité Universitätsmedizin, 13353 Berlin, Germany; thomas.meyer@charite.de; 5 Ambulanzpartner Soziotechnologie APST GmbH, 13353 Berlin, Germany; 6 Department of Neurology, Hannover Medical School, 30625 Hannover, Germany; dengler.reinhard@mh-hannover.de (R.D.); petri.susanne@mh-hannover.de (S.P.)

**Keywords:** amyotrophic lateral sclerosis, motor neuron disease, pain, pharmacotherapy, daily functions

## Abstract

Background: Although pain is common in amyotrophic lateral sclerosis (ALS) and an effectively treatable symptom, it is widely under-recognized and undertreated. This study investigates epidemiological and clinical characteristics of pain, its impact and pharmacological treatment in ALS patients. In addition, opportunities for further optimization of pain therapy need to be identified. Methods: Patients from three German ALS outpatient clinics were asked to complete the Brief Pain Inventory and the ALS Functional Rating Scale—Extension and to participate in semi-structured telephone interviews. Results: Of the 150 study participants, 84 patients reported pain. Pain occurred across all disease stages, predominantly in the neck, back and lower extremities. It was described with a broad spectrum of pain descriptors and mostly interfered with activity-related functions. Of the 84 pain patients, 53.8% reported an average pain intensity ≥4 on the numerical rating scale (NRS), indicating pain of at least moderate intensity, and 64.3% used pain medication. Irrespective of the medication type, 20.4% of them had no sufficient pain relief. Thirteen out of 30 patients without pain medication reported an average NRS value ≥4. Eleven of them—mainly in the context of high pain interference with daily functions—were supposed to benefit from adequate pain therapy. However, many patients had relevant concerns and misconceptions about pain therapy. Conclusion: Given the frequency, extent and multi-faceted impact of pain, it is necessary to systematically assess pain throughout the disease course. Potentials to optimize pain therapy were seen in the subset of patients with insufficient pain relief despite medication and in those patients without pain medication but high pain interference. However, there is a need to respond to patients’ barriers to pain therapy.

## 1. Introduction

Amyotrophic lateral sclerosis (ALS) is a devastating neurodegenerative disorder with rapidly progressive muscle wasting and paralysis. Most patients die within 3 to 5 years of onset due to respiratory complications and ventilatory failure [1].

Symptomatic treatment is the cornerstone of current clinical care and is centered on alleviating symptoms and enhancing patients’ remaining quality of life [2]. A recent survey on symptom management in patients with ALS demonstrated that there are still unmet medical needs and that pain is one of the symptoms that needs to be better addressed [3].

Studies focusing on pain in ALS report considerably variable prevalence, ranging from 40% to 85% [4,5,6,7,8]. The reported pain intensity is also highly variable, ranging from mild [5,6,9], to moderate [7,10] and up to severe [11].

There is consensus from population-based controlled studies that patients with ALS suffer more frequently from pain [5,6,12], have a higher pain intensity and have more pain-related functional impairments [6,12]. A recent study demonstrated that pain also worsens quality of life, and not only when it is severe [9].

Given the fact that pain is a frequent and bothering symptom in patients with ALS, it offers a clinically relevant target in optimizing symptom management.

However, in contrast to the clinical relevance of pain, there is no robust information on the effectiveness of the various treatments that are currently in clinical use for the management of pain in ALS [13,14]. Patients in several survey-based studies on pain in ALS had an average pain relief from analgesic medication between 58% and 71% on a 0 to 100% relief scale [5,7,10,12], suggesting that the treatment is quite effective on average. However, further research on the sufficiency of pain relief on an individual basis and with regard to certain drug classes or combinations could help to identify further opportunities to improve pharmacotherapeutic pain management in ALS patients.

There is ample evidence in the literature that a considerable proportion of ALS patients reporting pain do not receive any pain medication. This proportion ranges from 13% to 53% of the patients with pain between studies [4,6,7,10,12,15,16]. However, further analysis of this patient group was beyond the scope of the respective articles and thus clinically important questions regarding the patients’ clinical and pain-related characteristics remain unanswered.

Therefore, a study of a large cohort of ALS patients is needed to critically appraise ALS-related pain therapy and to characterize those patients who suffer from pain and do not receive any pain medication. This may help to identify patients with potential benefit from pain therapy. Further research is warranted to explore ALS patients’ concerns towards analgesic medication. This knowledge will help to overcome barriers leading to unrelieved symptom distress and may thus contribute to improving patient-centered pain management strategies.

This prospective multi-center study aimed to examine epidemiological and clinical characteristics of pain as well as its impact on daily living in patients with ALS. It further evaluated the pharmacological pain therapy and pain relief through certain drug classes and combinations on an individual patient basis. Patients with pain who did not receive any pain medication were further evaluated and characterized to identify patients who may benefit from pain therapy.

## 2. Methods

### 2.1. Patient Recruitment

This study was approved by the institutional ethics committee of the University of Magdeburg and was conducted between June 2015 and June 2019. Patients with ALS were consecutively recruited during clinical consultation through the outpatient clinic at the University of Magdeburg. Further patients were asked to participate in this study at the outpatient clinics of Hannover Medical School and Charité Universitätsmedizin Berlin. They were subsequently contacted by a project team at the University of Magdeburg.

Exclusion criteria were insufficient comprehension of the German language or severe cognitive impairment, evaluated by clinical judgement during medical consultation, rendering adequate answering of the survey impossible.

A total of 182 patients were recruited for this study and provided written informed consent before enrollment.

### 2.2. Patient Characteristics

All patients were diagnosed according to the revised El Escorial criteria [17]. The patients were staged according to the onset site of the first symptom as bulbar, upper or lower limb [18]. Additionally, the ALS Functional Rating Scale—Extension (ALSFRS-EX) was used to derive the King’s clinical staging system for ALS, which categorizes the progressive clinical impairment on the basis of clinical milestones throughout the course of the disease. The stages refer to the functional involvement of anatomical regions and the need for nutritional and respiratory support [19], for which we used provision as a proxy [20].

### 2.3. Telephone Interviews and Patient-Reported Assessment Instruments

All phone calls were conducted after making an appointment with the patient. During the contact, we collected demographic and clinical information such as disease duration and supportive therapy, e.g., placement of a percutaneous gastrostomy tube and implementation of non-invasive ventilation (NIV). Further questions were asked about pain and pain medication or possible barriers to using it. In addition, the patients were asked whether they suffered from muscle cramps. In case of specific pain problems, such as pain in the face, deeper questions were asked to find out more about the cause, e.g., mask-induced skin lesions or pressure ulcers. Therefore, the use, type of NIV interface and the tolerance of NIV were elicited.

For patients who were unable to conduct the interview due to pronounced bulbar symptoms or dyspnea, it was possible to conduct the interview with the help of a relative. During the phone calls, detailed notes of the responses to the questions were taken and analyzed descriptively.

Subsequently, questionnaires were sent home to the patients. To assess physical functioning, patients were asked to complete the disease-specific ALS Functional Rating Scale—Extension (ALSFRS-EX), which consists of 15 items in four subscales (i.e., bulbar, fine motor, gross motor and respiratory) and has been validated as a patient-reported measurement instrument in the German language [21,22]. The sum score ranges from 0 to 60 points with a maximum subscale score of 16 points for the bulbar, fine and gross motor subscales and 12 points for the respiratory subscale. Lower scores represent a worse condition. For pain assessment, the German version of the Brief Pain Inventory (BPI) was used as a patient-reported measure in its long form [23]. The BPI contains questions on pain intensity and interference of pain with daily functions, which are rated on scales from 0 to 10 points using a recall period of one week. In addition, the patients localize their pain on front and back body drawings and mark word descriptors of pain quality to describe the pain. The BPI also asks about pain-relieving factors and the percentage of medication-related pain relief on a scale from 0 to 100.

To categorize the different levels of pain intensity of the BPI, we used cut-point 3 for mild–moderate and cut-point 6 for moderate–severe pain referring to the average pain intensity scale [24]. The BPI interference scale for assessing pain-related functional impairment as one of the core measures in studies with chronic pain patients [25] was evaluated as an overall score, the so-called pain interference total score of the BPI (BPI-PITS, the average score of all 7 items). Additionally, items were grouped into an activity subdimension (BPI-WAW, average score of work, general activity and walking), and an affective subdimension (BPI-REM, average score of relations with others, enjoyment of life and mood). As pain interference with sleep can be assigned to both subdimensions, the item “sleep” was evaluated separately [26].

In accordance with previous literature, pain interference scores ≥7 were categorized as high [27].

Pain relief of at least 10% is regarded as minimal, values ≥30% are considered as a moderate degree of pain relief and values ≥50% are considered as substantial improvements due to pain medication [25].

The assessment instruments were completed by the patients or, if this was not possible, by their relatives or caregivers according to the patient’s information, and then returned.

### 2.4. Evaluation of Pain Patients without Pain Medication

Patients with pain who did not receive any pain medication were stratified according to their pain-related features. First, they were classified into patients with mild and at least moderate pain (defined as ≥4 on the numeric rating scale, NRS) referring to the average pain intensity scale. In the next step, we analyzed whether those patients with at least moderate pain intensity differed in their ALS-related clinical characteristics from those receiving pain medication, and whether patient-reported barriers to pharmacotherapeutic pain management were present in these patients. They were further characterized to identify patients with high pain interference with daily functions who may benefit from pain therapy.

### 2.5. Statistics

Statistics were calculated with IBM SPSS, Version 24 (IBM SPSS Statistics, Armonk, NY, USA). For demographic and clinical data, descriptive statistics (numbers and proportion or mean and standard deviation, as appropriate) were performed.

Correlations were assessed using Pearson’s and Spearman’s tests for normally and non-normally distributed data, respectively. For the interpretation of effect sizes, correlation values between 0.10 and 0.29 were considered weak, from 0.30 to 0.49 moderate and from 0.50 to 1.00 strong [28]. Comparisons between the groups were performed using Chi square test or Fisher’s exact test, as appropriate, for categorical data and the independent samples *t*-test or the Mann–Whitney U test for continuous data depending on the distribution of the data. The significance level was set at α ≤ 0.05, unless stated otherwise, and corrected for multiple comparisons using Bonferroni adjustments, if necessary.

## 3. Results

A detailed flow diagram of the recruitment including drop-outs is provided in Supplementary Figure S1.

Datasets of 150 patients were finally included in this study. Eighty-four patients had sufficient pain to fulfill the criteria of the BPI to complete the entire questionnaire. Accordingly, pain was prevalent in 56% of the patients. Patient-referred data of the patients with and without pain are reported in Table 1. Patients with pain were significantly younger (*p* = 0.037) than patients without pain and had a significantly lower ALSFRS-EX sum score (*p* = 0.003), indicating more physical impairment with significantly lower gross motor (*p* = 0.012) and fine motor (*p* = 0.003) subscores. Regarding the other demographical and clinical data, no significant differences were observed between the two groups.

Of the 84 pain patients, the majority (84.3%) attributed their pain to ALS, and approximately one quarter (27.4%) had experienced pain at the time when ALS was diagnosed.

### 3.1. Pain Intensity

**Figure 1:**
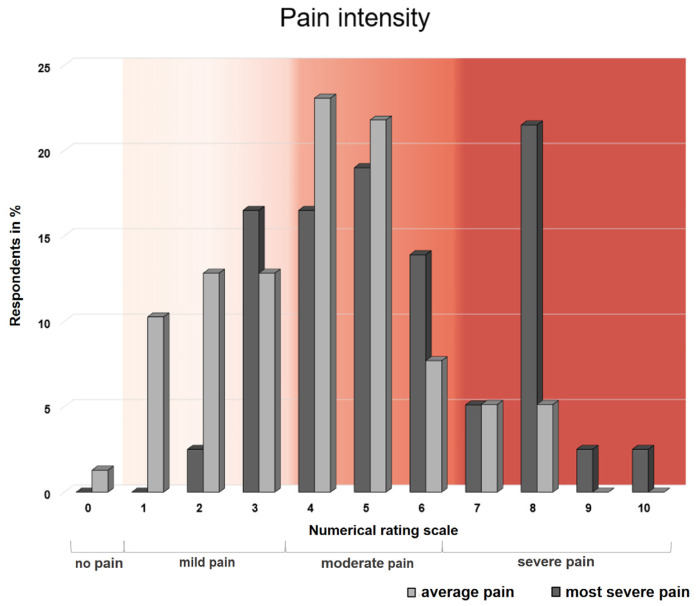
Pain intensity according to the Brief Pain Inventory (BPI) for the average pain in the past week (light gray bars) and for the most severe pain in the past week (dark gray bars). The number of respondents is given as percentages. The pain intensity is scaled from 0 (=no pain) to 10 (=strongest pain imaginable) points on the numerical rating scale. The increasing levels of the average pain intensity are highlighted in shades of red from lighter to darker.

The pain intensity ratings for the average and most severe pain in the past week are presented in Figure 1. The mean score for the average pain was 4.0 ± 1.9 on the NRS. Up to 53.8% of the pain patients suffered from an average NRS pain intensity score ≥4, with 10.2% of these patients presenting with severe pain. The average score for the most severe pain in the past week was 5.5 ± 2.0.

### 3.2. Locations of Pain

**Figure 2:**
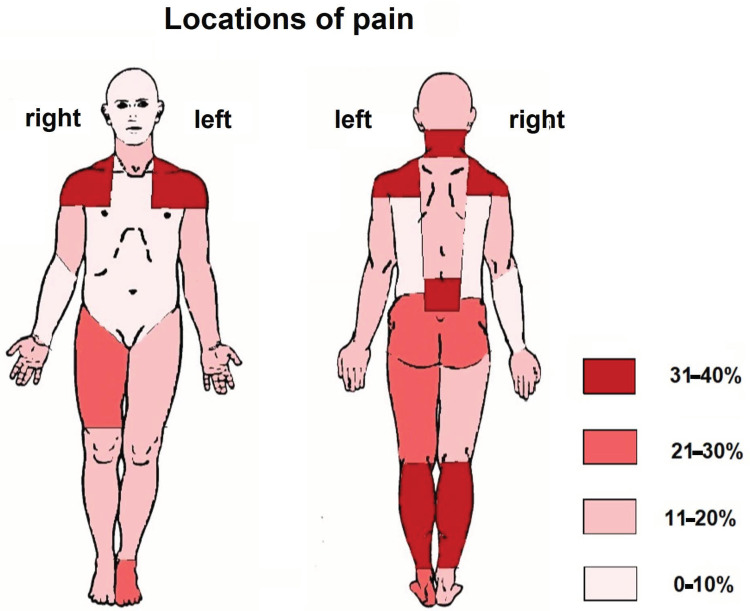
Representation of pain locations on front and back body diagrams indicating percentage frequency distributions for the patients with pain.

Pain locations are visualized on front and back body diagrams in Figure 2. The most frequently affected body regions were the lumbar region (36.7%), neck (34.6%), shoulder region (left 32.9% and right 31.6%) and calves (left 32.9% and right 30.4%), followed by the buttocks (27.8%) and proximal leg (right front side 25.3% and back side 20.3%).

Eight out of 26 patients (30.8%) with pain requiring NIV reported painful skin lesions over the nasal bridge due to the NIV mask, which corresponds to 9.5% of the 84 patients with pain. In six patients, these lesions were specified as pressure ulcers. Seven out of these eight patients used orofacial masks and one patient used a nasal mask. The extent of NIV use was 20 per 24 h in one patient, 10 h in two patients and 7 h in two further patients. Three patients reported that they had difficulty tolerating NIV, using it only intermittently and for a variable number of hours a day.

### 3.3. Pain Perception

**Figure 3:**
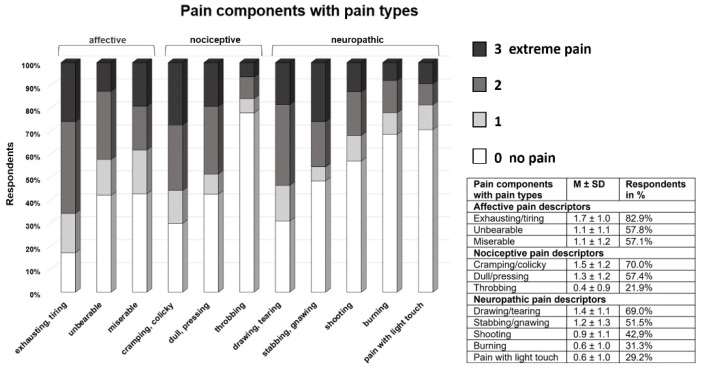
Descriptors of the pain sensations listed in the BPI with means (M) and standard deviations (SD) as well as the percentage of the respondents reporting the respective type of pain. The boxes of each stacked bar graph illustrate the response categories of the respective item from 0 = no pain to 3 = extreme pain. The height of the boxes shows the percentage of respondents who chose the respective response category. The items for pain descriptions are arranged within the respective pain components in descending order of frequency.

The patients’ ratings for the different pain qualities are depicted in Figure 3. We assigned the pain qualities listed in the BPI to the sensory and affective components of pain [30] and further distinguished between neuropathic and nociceptive pain descriptors.

When focusing on items representing the affective component of pain perception, most of the patients (82.9%) described their pain as “exhausting, tiring”, followed by “unbearable” (57.8%) and “miserable” (57.1%). Of the nociceptive pain descriptors, “cramping/colicky” ranks first (70%) and “dull, pressing” second (57.4%). The item “throbbing” was clearly less frequently chosen for pain description (21.9%). Among the neuropathic pain descriptors, “drawing, tearing” (69%), “shooting” (42.9%) and “stabbing, gnawing” (51.5%) were mentioned most frequently. The neuropathic pain qualities “burning” (31.3%) and “pain with light touch” (29.2%) were regarded as less appropriate for pain description. Patients selected on average 4.7 ± 2.6 terms to describe their pain.

In addition to the pain qualities in the BPI, patients were asked during the telephone interviews whether they suffered from muscle cramps in order to specify the item “cramping/colicky” of the BPI. This was confirmed by 69% of the patients.

### 3.4. Pain Interference with Daily Functions

**Figure 4:**
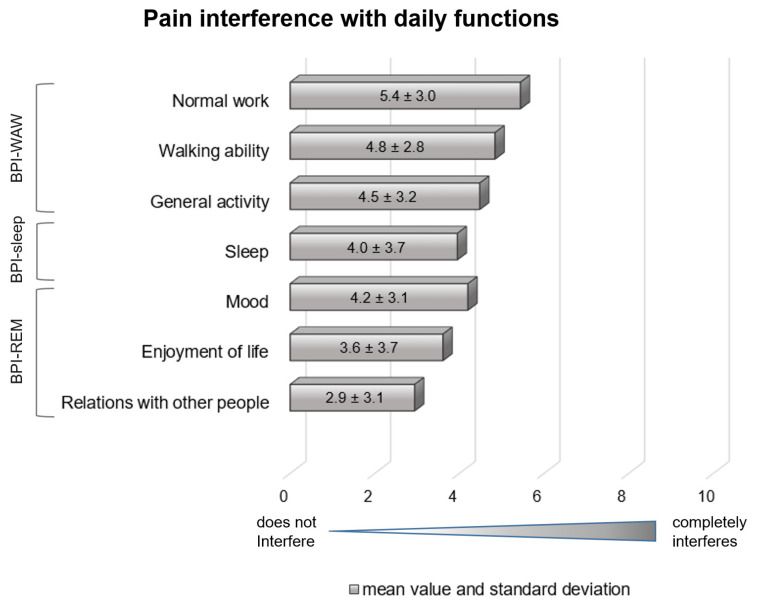
Daily functions impaired by pain, indicating the mean values on a scale from 0 (=does not interfere) to 10 (=completely interferes). The items are arranged within the respective BPI subdimensions of pain interference in descending order of the mean values. BPI = Brief Pain Inventory; REM = affective subdimension of the BPI; WAW = activity subdimension of the BPI.

Figure 4 visualizes the patients’ ratings for the pain interference with daily functions. Pain interfered with all aspects of daily living listed in the BPI, with activity-related functions (BPI-WAW) being most affected by pain. Regarding the items of the affective subdimension (BPI-REM), pain mostly interfered with “mood”. The pain interference total score of the BPI (BPI-PITS) reached a value of 4.2 ± 2.4.

### 3.5. Evaluation of Pharmacological Pain Therapy and the Patients’ Perceptions of Pain Relief

The pain-related pharmacotherapy is presented in Table 2 and refers for comparative purposes with other studies to all 84 patients with pain. As cramps and spasticity can be a source of pain, the respective pharmacotherapeutic treatments are also reported.

Of the 84 pain patients, 54 patients (64.3%) received pain medication. Non-opioid analgesics were most commonly used (53.6%), mainly non-steroidal anti-inflammatory drugs (NSAIDs) and metamizole. Opioids were the second most frequently used drug class (19%), with low-potency opioids being slightly more often used than high-potency opioids. A smaller proportion of the patients received coanalgesics such as anticonvulsants (12%) or antidepressants (6%). A further 13 patients received antidepressants with analgesic properties for other indications than pain relief (e.g., sialorrhea, depressive mood disorder). Seventeen patients (20.2%) received treatment for cramps, in approximately equal parts magnesium and quinine sulfate. ALS-related spasticity was treated in seven of the pain patients (8.3%), mainly with baclofen.

Table 3 outlines the patients’ perceptions of medication-related pain relief considering certain drug combinations. The average pain relief across the different pain medications was 45.4% ± 27.4. The two patient groups treated with coanalgesics (antidepressants or anticonvulsants) and high-potency opioids alone or in combination experienced on average substantial pain relief. In the other two patient groups taking non-opioid analgesics or low-potency opioids alone or in combination, the pain relief was moderate on average.

In terms of clinically relevant degrees of pain relief, 12 patients (22.2%) had moderate and 27 patients (50%) substantial pain relief. Eleven patients (20.4%) had no sufficient pain relief (defined as pain relief <30%). With regard to the type of medication in use, the proportion of patients with no sufficient pain relief was 33.4% for coanalgesics, 18.8% for non-opioid analgesics, 20% for low-potency opioids and 16.7% for high-potency opioids.

Of the 84 pain patients, 34 patients (40.5%) stated that pain medication provided pain relief. Other frequently reported non-pharmacological pain relieving factors were resting (46.4%) as well as physiotherapy and movement (36.9%). In 23 of the patients (27.4%), warm compresses improved pain.

### 3.6. Characterization of the Pain Patients without Pain Medication

Of the 84 patients with pain, 30 patients (35.7%) did not receive any pain medication and were further stratified based upon their ratings of pain intensity and pain-related impairment. First, patients were grouped into those with mild pain (defined as NRS < 4) and those with moderate to severe pain intensity (defined as NRS ≥ 4) referring to the average pain intensity scale. Accordingly, 17 patients (56.7%) had mild pain and 13 patients (43.3%) at least moderate pain.

Demographic and clinical data of the 13 patients reporting an NRS value ≥4 were compared to pain patients with pain medication (Table 4). A striking finding was the significantly shorter time since diagnosis in the pain patients without pain medication (*p* = 0.001). This was accompanied by a markedly lower physical impairment reflected in a significantly higher ALSFRS-EX sum score (*p* = 0.048) and gross motor subscore (*p* = 0.006).

**Figure 5:**
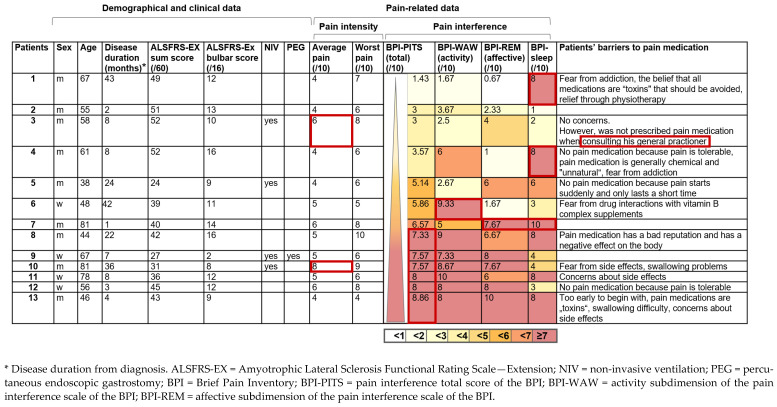
Characteristics of the 13 patients who did not receive any pain medication and who reported an average NRS value ≥4. The figure summarizes the demographic, clinical and pain-related data as well as the patient-reported barriers to pain medication with the patients being arranged according to the BPI pain interference total score (BPI-PITS) in ascending order. Pain interference scores ≥7 were categorized as high and are highlighted in dark red. For visualization of the level of pain interference referring to the scores between 0 and 6, an additional color scale from white to shades of yellow and orange was used. The findings highlighted by dark red boxes prompted us to assume that the respective patients may benefit from adequate pain therapy.

The 13 patients without pain medication and an NRS value ≥ 4 were characterized with regard to their demographic, clinical and pain-related data on an individual basis (see Figure 5) in order to identify patients who may benefit from pain therapy. A high overall pain interference or a high pain interference with certain aspects of daily functions prompted us to assume that the respective patient may benefit from adequate pain therapy. Accordingly, six patients presented with a high overall pain interference (patients 8–13) and a further four patients presented with a high pain-related impairment of certain components of daily functioning, such as high activity interference seen in patient 6 or high pain interference with sleep seen in patients 1, 4 and 7. In patient 7, who reported a maximum pain interference with sleep, a high affective interference was also observed. In summary, 10 patients suffered from high pain-related impairments and may thus profit from adequate pain therapy. In another patient (patient 3), pain therapy should be re-considered. This patient reported an average NRS value of 6 corresponding to the higher value range of moderate pain intensity, and had a moderate pain interference with daily functions. He mentioned that he consulted a general practitioner, but was not prescribed any pain medication. This demonstrates the patient’s need for pain relief. Taken together, pain therapy should be considered in 11 patients.

When asking the patients about the reasons why they did not take any pain medication, 8 out of these 11 in whom pain therapy should be considered described concerns about pain medication and provided explanations about why they were reluctant or unwilling to take pain medication (see Figure 5). The most frequently mentioned patient-reported barriers were side effects (patients 8, 10, 11, 13), and the belief that pain medications are “toxins” (patients 1, 13), “chemical” (patient 4) and have a bad reputation (patient 8). Few patients reported that they had a fear of addiction (patients 1, 4), swallowing difficulties (patients 13, 10) and/or that their pain was tolerable (patients 4, 12). One patient said that it was “too early to begin with pain medication” (patient 13).

## 4. Discussion

This multi-center study in a well-characterized cohort of 150 patients with ALS showed that pain was prevalent in 56% of patients and can occur at any stage of the disease. Eighty-five percent of the patients attributed their pain directly to ALS. Pain was most frequently prevalent in the lumbar region, neck, shoulder region, buttocks and legs. The pain descriptor profile included a variety of affective and sensory pain descriptor terms. A substantial subset of the patients with pain (53.8%) suffered from an average NRS pain intensity score ≥4. Pain had a negative impact on different aspects of daily living, mostly on activity-related functions. Of the 84 pain patients, about two-thirds received pain medication, most commonly non-opioid analgesics (53.6%), followed by opioids (19%) and coanalgesics (18%). Half of the patients reported substantial and 22.2% moderate pain relief, whereas 20.4% of the patients had no sufficient pain relief. Of the 30 pain patients without pain medication, 13 reported an NRS score ≥4 for their average pain. They had a significantly shorter time since diagnosis and a better physical status than those with pain medication. We suspect that 11 of these 13 patients, mostly with high pain interference with daily functions, may benefit from adequate pain therapy. When asked about the reasons why they did not take any pain medication, many of those patients described concerns about pain therapy, mainly misconceptions about analgesics.

### 4.1. Pain Characteristics and Impact

The finding that the majority of our pain patients attributed their pain to ALS seems to be surprising because pain is a non-motor symptom and not primarily associated with ALS. However, our observation is consistent with previously published data [10] and emphasizes the clinical relevance of pain as an accompanying symptom in patients with ALS.

Our study demonstrates that pain is present at any stage of the disease, which is in agreement with a previous study that pointed to pain as a common symptom in ALS regardless of the disease stage [31]. Our study confirms that in up to one third of the patients, pain was already present when ALS was diagnosed [7,10]. Thus, a careful and systematic assessment of pain is needed from the time of the diagnosis of ALS throughout the course of the disease.

The pain locations most frequently mentioned in our study cohort are similar to previous findings [5,6,7,10,11] and indicate pain of musculoskeletal origin with a predominant axial distribution [18,32].

Up to one third of our pain patients requiring NIV had painful skin lesions on the nasal bridge, including pressure ulcerations due to NIV, with half of them being poorly adapted to NIV. Indeed, this painful complication is a common reason for poor adaptation or non-invasive ventilation failure [33] and should be proactively managed because NIV can improve quality of life and survival and has become an important cornerstone of symptomatic treatment in patients with ALS [34,35]. In such cases, the use of a total full-face mask is recommended, which covers the whole face, but has the disadvantage of greater dead space and leakage [35]. Other masks such as nasal masks and minimal full face masks that do not press on the nasal bridge can help to prevent further skin irritation and air leaks.

The pain descriptor profile of our patients included multiple affective and sensory pain descriptors and reached an average number of almost five descriptor terms per patient. Previous studies congruently observed the use of a variety of pain descriptor terms in patients with ALS [10,36], indicating the multifactorial nature of pain in ALS [10]. In a case–control observational study, the greater number of co-existing pain types observed in ALS patients reached statistical significance compared to healthy controls [5]. Clinicians should carefully assess the individual pain descriptor profile in ALS patients suffering from pain. This may help to draw conclusions on relevant pain dimensions and potentially underlying pain mechanisms [37,38,39] and to develop a comprehensive treatment plan for the individual patient.

Clinical studies often categorize the patients’ ratings of their pain into mild, moderate and severe pain. According to a literature review, there is a wide range of cut-points for these categories on the numeric rating scale depending on the disease and mechanism of pain [40]. The optimal cut-point scheme in patients with ALS remains to be determined. Most pain studies in ALS use cut-point 3 to discriminate mild from moderate pain [33]. In accordance, we used cut-point 3 for mild–moderate and additionally cut-point 6 for moderate–severe pain. These cut-points were proposed for patients with chronic non-cancer pain and refer to the average pain intensity scale, which better reflects the degree of pain interference with daily living in these patients [24].

The average pain intensity in our study cohort can be considered moderate and is comparable to previous studies, as was the moderate pain interference total score of the BPI as a measure for the average degree to which pain interferes with daily functions [7,10]. Pain mostly interfered with activity-related functions of daily living and can thus contribute to further deterioration in the patients’ daily functioning beyond the impact of ALS itself.

Even though quality of life is undoubtedly a different construct than what is measured by pain interference according to the BPI, there is a certain analogy in the predominant impact of pain on physical aspects of the respective construct. A recent study in ALS patients demonstrated that pain influences physical quality of life more than psychological quality of life [9].

### 4.2. Pharmacotherapeutic Pain Management

In our study, the high proportion of patients taking non-opioid analgesics is comparable with previous literature [4,15], as was the low percentage of patients taking coanalgesics [6,15]. The proportion of pain patients taking opioids lies in the middle range of previously reported data [4,6,10,15,16].

When comparing the proportion of patients stating pain medication to be a pain-relieving factor, the percentage of patients in our study was higher than in previous literature [5]; however, the average medication-related pain relief in percent was lower than in other series of ALS patients with pain [5,7,10,12]. The present study additionally evaluated the magnitude of pain relief for certain drug classes/combinations in terms of clinically important degrees of pain relief with the respective benchmarks for moderate (≥30%) and substantial pain relief (≥50%) [25]. This allows for a more meaningful interpretation of pain relief on an individual basis. Accordingly, half of our patients had substantial pain relief and approximately one quarter had moderate pain relief. However, about one fifth of our patients had no sufficient pain relief, which was irrespective of the medication in use and indicates the need to re-evaluate pain treatment. This represents a relevant potential to optimize pain therapy in this subset of patients.

Most studies on pain in ALS that evaluated pharmacotherapeutic management in their patients did not provide any data on those pain patients who did not receive pain therapy, although these patients presented a relevant subgroup of the respective pain patient cohorts [4,7,10,12,16]. A Swedish 3-year observational study reported that almost all patients with a pain intensity rating ≥4 on the NRS received some form of pain medication [41]. Another study from Germany in 46 ALS patients, of whom 78% suffered from pain, specified that 19% of the patients with mild pain used some kind of pain therapy, against 87% of those with moderate to severe pain [6].

In the present study, we further characterized the patients with pain who did not receive any pain medication and stratified them based upon their ratings of pain intensity and pain interference to identify those patients who may benefit from pain therapy. Generally, chronic pain patients with mild pain can be expected to have their pain adequately controlled, while patients with moderate or severe pain need a treatment reevaluation [24]. However, reports of pain intensity need to be interpreted in the context of other measures, such as functioning and quality of life [42]. In our study, 13 patients (43.3%) out of the 30 pain patients without pain medication reported an NRS score ≥4. They had a significantly shorter time since diagnosis accompanied by a significantly better overall functional status compared to the pain patients with pain medication. This was an unexpected finding. We assume that those patients who were confronted more recently with the diagnosis of ALS and thus had a better preserved functional status probably considered other issues to be much more important than their pain during medical consultation. A review on pain in ALS suggested that patients may be reluctant to report their pain out of fear to distract the physician from treating the primary consequences of the disease [32]. This may have also played a role in some of our patients, which may have been fostered by a noticeably short time since diagnosis. Noteworthy, in the subgroup of untreated pain patients with an NRS value ≥4, the bulbar and respiratory function was comparable to those patients using pain medication, and the gross motor function was even better preserved. Differences in supportive therapies with PEG and NIV were not found between both groups. Thus, disease-related impairments, such as dysphagia, the need to administer medication via appropriate alternative routes including enteral tube feeding, ventilatory insufficiency and severe general muscle weakness with increased risk of falling, which can make pharmacotherapeutic pain management in ALS more complex, have obviously not contributed to the undertreatment of pain in this patient group.

We suspect that 11 of these 13 patients, who mostly experienced high pain interference with daily functions, may profit from appropriate pain management. However, we found relevant patient-reported barriers to pain therapy in many of them. We assume that these barriers may have essentially contributed to the underreporting of pain in many of these patients or prevented them from wanting to mention their pain during consultations with their doctors.

Our study showed that the barriers to pain therapy were mainly misconceptions about analgesics. Careful exploration of these barriers offers the treating physician an opportunity to appropriately address these concerns within a patient-centered interaction to help to overcome barriers contributing to unrelieved symptom distress and to propose viable options for the respective patient to relieve pain-related suffering.

Although this study focused on pharmacotherapeutic pain management, the management of pain should involve a multiprofessional approach and consider suitable non-pharmacological treatment options, which may be valuable and successful in the management of chronic pain [43]. A close interaction and collaboration of the treating ALS specialist with a physical and/or occupational therapist, a clinical psychologist, rehabilitation professionals and a pain physician, as needed and depending on local resources and available expertise, will help to respond appropriately to pain-related impairments [27,32,43].

Further research on pharmacological and non-pharmacological treatments in different types of ALS-associated pain conditions is warranted to support clinical decision-making and to improve the management of pain in patients with ALS.

### 4.3. Limitations

A potential limitation of this study is that the categorizations of the different levels of pain intensity and of pain interference with daily functioning were derived from studies on chronic pain and have not been verified in patients with ALS. Moreover, the patients’ tolerability of pain and preferences for treatment as relevant factors involved in analgesic decision-making were not accounted for in our study. Lastly, our data refer to patients who, in their vast majority, regularly attended medical consultations in specialized ALS outpatient clinics, which may limit the generalizability of our findings.

## 5. Conclusions

Given the frequency, the extent and the multi-faceted impact of pain on daily living in ALS patients, it is necessary to systematically assess pain throughout the course of the disease to meet the needs of ALS patients with pain. Effective treatment of pain has the potential to improve the remaining quality of life, which is of utmost importance when survival time is so limited. This study identified opportunities to optimize pain therapy in a subset of patients with insufficient medication-related pain relief and in an equally large proportion of pain patients without pain medication who mostly experienced severe pain interference with daily functions. In the latter, we found relevant patient-reported barriers to pain therapy, which should be carefully evaluated and addressed appropriately. This can help to overcome barriers contributing to unrelieved symptom distress and may thus contribute to improving pain management strategies in individual patients with ALS.

## Figures and Tables

**Table 1:** Demographic and clinical data of ALS patients with and without pain (mean ± SD or number of patients (%), as appropriate).

	Patients with Pain, *n* = 84 (56%)	Patients without Pain, *n* = 66 (44%)	*p*-Value
Gender (female/male)	24/60 (28.6/71.4%)	20/46 (30.3/69.7%)	0.817
Patient age	61.2 ± 11.8	66.6 ± 8.0	**0.037**
Disease duration from diagnosis in months	33.5 ± 38.5	37.7 ± 51.3	0.569
Symptom onset			0.135
Bulbar	14 (16.7%)	19 (28.8%)	
Upper limb	37 (44%)	21 (31.8%)	
Lower limb	31 (36.9%)	22 (33.3%)	
King‘s clinical staging			0.065
Stage 1: Symptom onset/functional involvement of first region	0	0	
Stage 2A: Diagnosis	7 (8.3%)	5 (7.6%)	
Stage 2B: Functional involvement of a second region	8 (9.5%)	16 (24.2%)	
Stage 3: Functional involvement of a third region	39 (46.4%)	23 (34.8%)	
Stage 4A: Need for gastrostomy	4 (4.8%)	7 (10.6%)	
Stage 4B: Need for respiratory support (NIV)	26 (31%)	15 (22.7%)	
ALSFRS-EX			
Sum score	36.6 ± 12.9	42.6 ± 10.8	**0.003**
Bulbar subscore	11.6 ± 4.8	11.23 ± 3.4	0.344
Fine motor subscore	7.7 ± 4.3	10.0 ± 4.9	**0.003**
Gross motor subscore	8.2 ± 5.0	10.2 ± 4.6	**0.012**
Respiratory subscore	9.1 ± 3.2	10.2 ± 2.8	0.038
Disease progression rate *	0.9 ± 1.9	0.75 ± 0.9	0.639

NIV = Non-invasive ventilation. * Disease progression rate was calculated as (60-sum of ALSFRS-EX)/disease duration from symptom onset to investigation date in months (adapted from [22,29]). Significant *p*-values are boldfaced. Referring to the ALSFRS-EX subscales, Bonferroni-adjusted *p*-values 0.05/4 = 0.0125 were deemed statistically significant.

**Table 2:** Data referring to the medication of the 84 pain patients regarding the treatment for pain as well as the treatment for cramps and spasticity. Values are given as number of patients (%).

Pharmacotherapy *	Patients with Pain
**Treatment for pain** (*n* = 54 (64.3%))	
**Non-opioid analgesics** (*n* = 45 (53.6%))	
Non-steroidal anti-inflammatory drugs (NSAID) Metamizole Paracetamol COX-2 inhibitor drugs	21 22 8 5
**Opioid analgesics** (*n* = 16 (19%)) Low-potency opioids Tramadol Tilidine High-potency opioids Morphine Hydromorphone Fentanyl transdermal patch Buprenorphine sublingual tablet	4 6 1 2 2 1
**Tricyclic antidepressants** (*n* = 5 (6.0%)) **	5
**Anticonvulsants** (*n* = 10 (12.0%)) Gabapentin Pregabalin	2 8
**Treatment for cramping** (*n* = 17 (20.2%))	
Magnesium Quinine sulfate	8 11
**Treatment for spasticity** (*n* = 7 (8.3%))	
Baclofen Tolperisone Intramuscular injections of botulinum toxin	5 1 2

* This table does not consider any combination of therapies. Thus, the total percentage of patients is higher than 100%. ** Antidepressants refer to their use for analgesic purposes.

**Table 3:** Pain-related pharmacotherapy considering drug combinations in the 54 pain patients with pain medication. The patients’ perception of medication-related pain relief is presented as an average value and in terms of clinically relevant degrees of pain relief for the respective type of medication (no pain relief: <10%; minimal pain relief: ≥10 and ˂30%; moderate pain relief: ≥30%; substantial pain relief ≥50%). Values are given as mean ± SD or number of patients (%), as appropriate.

Pain-Related Pharmacotherapy	Patients (*n* = 54)	Average Pain Relief in %	Degree of Pain Relief				
		M ± SD	No	Minimal	Moderate	Substantial	No Information
**Treatment with coanalgesics**	6 (11.1%)	55 ± 39.4	1 (16.7%)	1 (16.7%)	0	4 (66.7%)	
Antidepressant alone	3						
Anticonvulsant alone	3						
**Treatment with non-opioid analgesics**	32 (59.3%)	43.8 ± 28	6 (18.8%)	0	6 (18.8%)	17 (53.1%)	3 (9.4%)
Non-opioid analgesic alone	23						
Combination of non-opioid analgesics	3						
Non-opioid analgesic + antidepressant	2						
Non-opioid analgesic + anticonvulsant	4						
**Opioid treatment with low-potency opioids**	10 (18.5%)	36.7 ± 17.3	0	2 (20%)	5 (50%)	2 (20%)	1 (10%)
Low-potency opioid alone	1						
Low-potency opioid + anticonvulsant	1						
Low-potency opioid + non-opioid analgesic	7						
Low-potency opioid + non-opioid analgesic + anticonvulsant	1						
**Opioid treatment with high-potency opioids**	6 (11.1%)	56.7 ± 23.4	0	1 (16.7%)	1 (16.7%)	4 (66.7%)	
High-potency opioid alone	1						
High-potency opioid + non-opioid analgesic	4						
High-potency opioid + non-opioid analgesic + anticonvulsant	1						

Antidepressants refer to their use for analgesic purposes.

**Table 4:** Demographic and clinical data of the ALS patients without pain medication and at least moderate pain (NRS ≥4) compared to those patients with pain medication (mean ± SD or number of patients (%), as appropriate).

	Pain Patients without Pain Medication and NRS ≥4 (*n* = 13)	Pain Patients with Pain Medication (*n* = 54)	*p*-Value
Gender (female/male)	4/9 (30.8%/69.2%)	18/36 (33.3%/66.7%)	0.569
Patient age	60.0 ± 14.2	61.9 ± 12.5	0.625
Disease duration from diagnosis in months	16.0 ± 15.6	41.5 ± 43.9	**0.001**
ALSFRS-EX			
Sum score	40.9 ± 9.3	33.0 ± 13.1	**0.048**
Bulbar subscore	11.1 ± 3.7	11.2 ± 4.8	0.906
Fine motor subscore	9.4 ± 4.2	6.6 ± 4.5	0.047
Gross motor subscore	10.3 ± 3.7	6.6 ± 4.7	**0.006**
Respiratory subscore	10.1 ± 1.4	8.8 ± 3.2	0.034
PEG	1 (7.7%)	12 (23.1%)	0.215
NIV	3 (23.1%)	17 (32.7%)	0.502

Significant *p*-values are boldfaced. Referring to the ALSFRS-EX subscales, Bonferroni-adjusted *p*-values 0.05/4 = 0.0125 were deemed statistically significant.

## Data Availability

Data are contained within the article.

## Supplementary Materials

The following are available online at https://www.mdpi.com/article/10.3390/jcm10194552/s1, Figure S1: Flow diagram of recruitment scheme.

## References

[1] Ringel S.P., Murphy J.R., Alderson M.K., Bryan W., England J.D., Miller R.G., Petajan J.H., Smith S.A., Roelofs R.I., Ziter F. (1993). The natural history of amyotrophic lateral sclerosis. Neurology.

[2] Jenkins T.M., Hollinger H., McDermott C.J. (2014). The evidence for symptomatic treatments in amyotrophic lateral sclerosis. Curr. Opin. Neurol..

[3] Nicholson K., Murphy A., McDonnell E., Shapiro J., Simpson E., Glass J., Mitsumoto H., Forshew D., Miller R., Atassi N. (2018). Improving symptom management for people with amyotrophic lateral sclerosis. Muscle Nerve.

[4] Ganzini L., Johnston W.S., Hoffman W.F. (1999). Correlates of suffering in amyotrophic lateral sclerosis. Neurology.

[5] Wallace V.C.J., Ellis C.M., Burman R., Knights C., Shaw C.E., Al-Chalabi A. (2014). The evaluation of pain in amyotrophic lateral sclerosis: A case controlled observational study. Amyotroph. Lateral Scler. Frontotemporal. Degener.

[6] Hanisch F., Skudlarek A., Berndt J., Kornhuber M.E. (2015). Characteristics of pain in amyotrophic lateral sclerosis. Brain Behav..

[7] Lopes L.C.G., Galhardoni R., Silva V., Jorge F.M.H., Yeng L.T., Callegaro D., Chadi G., Teixeira M.J., Ciampi de Andrade D. (2018). Beyond weakness: Characterization of pain, sensory profile and conditioned pain modulation in patients with motor neuron disease: A controlled study. Eur. J. Pain.

[8] Wicks P. (2012). Reassessing received wisdom in ALS–pain is common when studied systematically. Eur. J. Neurol..

[9] Edge R., Mills R., Tennant A., Diggle P.J., Young C.A., TONiC Study Group (2020). Do pain, anxiety and depression influence quality of life for people with amyotrophic lateral sclerosis/motor neuron disease? A national study reconciling previous conflicting literature. J. Neurol..

[10] Stephens H.E., Lehman E., Raheja D., Yang C., Walsh S., Mcarthur D.B., Simmons Z. (2016). Pain in amyotrophic lateral sclerosis: Patient and physician perspectives and practices. Amyotroph. Lateral Scler. Frontotemporal Degener..

[11] Pizzimenti A., Aragona M., Onesti E., Inghilleri M. (2013). Depression, pain and quality of life in patients with amyotrophic lateral sclerosis: A cross-sectional study. Funct. Neurol..

[12] Chiò A., Canosa A., Gallo S., Moglia C., Ilardi A., Cammarosano S., Papurello D., Calvo A. (2012). Pain in amyotrophic lateral sclerosis: A population-based controlled study. Eur. J. Neurol..

[13] Ng L., Khan F., Young C.A., Galea M. (2017). Cochrane Neuromuscular Group. Symptomatic treatments for amyotrophic lateral sclerosis/motor neuron disease. Cochrane Database Syst. Rev..

[14] Brettschneider J., Kurent J., Ludolph A. (2013). Cochrane Neuromuscular Group. Drug therapy for pain in amyotrophic lateral sclerosis or motor neuron disease. Cochrane Database Syst. Rev..

[15] Moisset X., Cornut-Chauvinc C., Clavelou P., Pereira B., Dallel R., Guy N. (2016). Is there pain with neuropathic characteristics in patients with amyotrophic lateral sclerosis? A cross-sectional study. Pall. Med..

[16] Ishida N., Hongo S., Kumano A., Hatta H., Zakoji N., Hirutani M., Yamamoto Y., Aono H., Tuigi M., Suzuki R. (2018). Relationship between pain and functional status in patients with amyotrophic lateral sclerosis: A multicenter cross-sectional study. J. Palliat. Med..

[17] Brooks B.R., Miller R.G., Swash M., Munsat T.L. (2000). World Federation of Neurology Research Group on Motor Neuron Diseases. El Escorial revisited: Revised criteria for the diagnosis of amyotrophic lateral sclerosis. Amyotroph. Lateral Scler. Other Motor Neuron Disord..

[18] Turner M.R., Wicks P., Brownstein C.A., Massagli M.P., Toronjo M., Talbot K., Al-Chalabi A. (2011). Concordance between site of onset and limb dominance in amyotrophic lateral sclerosis. J. Neurol. Neurosurg. Psychiatry.

[19] Roche J.C., Rojas-Garcia R., Scott K.M., Scotton W., Ellis C.E., Burman R., Wijesekera L., Turner M.R., Leigh P.N., Shaw C.E. (2012). A proposed staging system for amyotrophic lateral sclerosis. Brain.

[20] Balendra R., Jones A., Jivraj N., Knights C., Ellis C.M., Burman R., Turner M.R., Leigh P.N., Shaw C.E., Al-Chalabi A. (2014). Estimating clinical stage of amyotrophic lateral sclerosis from the ALS Functional Rating Scale. Amyotroph. Lateral Scler. Front. Degener..

[21] Wicks P., Massagli M.P., Wolf C., Heywood J. (2009). Measuring function in advanced ALS validation of ALSFRS-EX extension items. Eur. J. Neurol..

[22] Abdulla S., Vielhaber S., Körner S., Machts J., Heinze H.J., Dengler R., Petri S. (2013). Validation of the German version of the extended ALS functional rating scale as a patient-reported outcome measure. J. Neurol..

[23] Radbruch L., Loick G., Kiencke P., Lindena G., Sabatowski R., Grond S., Lehmann K.A., Cleeland C.S. (1999). Validation of the German version of the Brief Pain Inventory. J. Pain Symptom Manag..

[24] Brown K.E., Swift I., Spark M.J. (2012). Pain severity cut-points and analgesic use by community-dwelling people for chronic pain. J. Pharm. Pract. Res..

[25] Dworkin R.H., Turk D.C., Wyrwich K.W., Beaton D., Cleeland C.S., Farrar J.T., Haythornthwaite J.A., Jensen M.P., Kerns R.D., Ader D.N. (2008). Interpreting the clinical importance of treatment outcomes in chronic pain clinical trials: IMMPACT recommendations. J. Pain.

[26] Cleeland C.S. (2009). The Brief Pain Inventory User Guide. https://www.mdanderson.org/documents/Departments-and-Divisions/Symptom-Research/BPI_UserGuide.pdf.

[27] Miettinen T., Kautiainen H., Mäntyselkä P., Linton S.J., Kalso E. (2019). Pain interference type and level guide the assessment process in chronic pain: Categorizing pain patients entering tertiary pain treatment with the Brief Pain Inventory. PLoS ONE.

[28] Cohen J. (1988). Statistical Power Analysis for the Behavioural Sciences.

[29] Ellis C.M., Simmons A., Jones D.K., Bland J., Dawson J.M., Horsfield M.A., Williams SCLeigh P.N. (1999). Diffusion tensor MRI assesses corticospinal tract damage in ALS. Neurology.

[30] Fillingim R.B., Loeser J.D., Baron R., Edwards R.R. (2016). Assessment of chronic pain: Domains, methods, and mechanisms. J. Pain.

[31] Rivera I., Ajroud-Driss S., Casey P., Heller S., Allen J., Siddique T., Sufit R. (2013). Prevalence and characteristics of pain in early and late stages of ALS. Amyotroph. Lateral Scler. Front. Degener..

[32] Handy C.A., Krudy C., Boulis N., Federici T. (2011). Pain in amyotrophic lateral sclerosis: A neglected aspect of disease. Neurol. Res. Int..

[33] Chiò A., Mora G., Lauria G. (2017). Pain in amyotrophic lateral sclerosis. Lancet Neurol..

[34] Bourke S.C., Tomlinson M., Williams T.L., Bullock R.E., Shaw P.J., Gibson G.J. (2006). Effects of non-invasive ventilation on survival and quality of life in patients with amyotrophic lateral sclerosis: A randomized controlled trial. Lancet Neurol..

[35] Dorst J., Ludolph A.C. (2019). Non-invasive ventilation in amyotrophic lateral sclerosis. Ther. Adv. Neurol. Disord..

[36] Pagnini F., Lunetta C., Banfi P., Rossi G., Fossati F., Marconi A., Castelnuovo G., Corbo M., Molinari E. (2012). Pain in amyotrophic lateral sclerosis: A psychological perspective. Neurol. Sci..

[37] Gilron I., Tu D., Holden R.R. (2013). Sensory and affective pain descriptors respond differentially to pharmacological interventions in neuropathic conditions. Clin. J. Pain.

[38] Vardeh D., Mannion R.J., Woolf C.J. (2016). Towards a mechanism-based approach to pain diagnosis. J. Pain.

[39] Rudnicki S., McVey A.L., Jackson C.E., Dimachkie M.M., Barohn R.J. (2015). Symptom management and end of life care. Neurol. Clin..

[40] Woo A., Lechner B., Fu T., Wong C.S., Chiu N., Lam H., Pulenzas N., Soliman H., DeAngelis C., Chow E. (2015). Cut points for mild, moderate, and severe pain among cancer and non-cancer patients: A literature review. Ann. Palliat. Med..

[41] Sandstedt P., Littorin S., Johansson S., Gottberg K., Ytterberg C., Kierkegaard M. (2018). Disability and contextual factors in patients with amyotrophic lateral sclerosis-A three-year observational study. J. Neuromuscul. Dis..

[42] Sullivan M.D., Ballantyne J.C. (2016). Must we reduce pain intensity to treat chronic pain?. Pain.

[43] El-Tallawy S.N., Nalamasu R., Salem G.I., LeQuang J.A.K., Pergolizzi J.V., Christo P.J. (2021). Management of musculoskeletal pain: An update with emphasis on chronic musculoskeletal pain. Pain Ther..

