# Supplementary material for: A Multi-Center Cohort Study on Characteristics of Pain, Its Impact and Pharmacotherapeutic Management in Patients with ALS
Source: J Clin Med. 2021 Sep 30;10(19):4552. doi: 10.3390/jcm10194552 (PMC8509485; doi:10.3390/jcm10194552)
Supplement: Supplementary file 1 [file jcm-10-04552-s001.zip › jcm-1380159-supplementary.pdf]

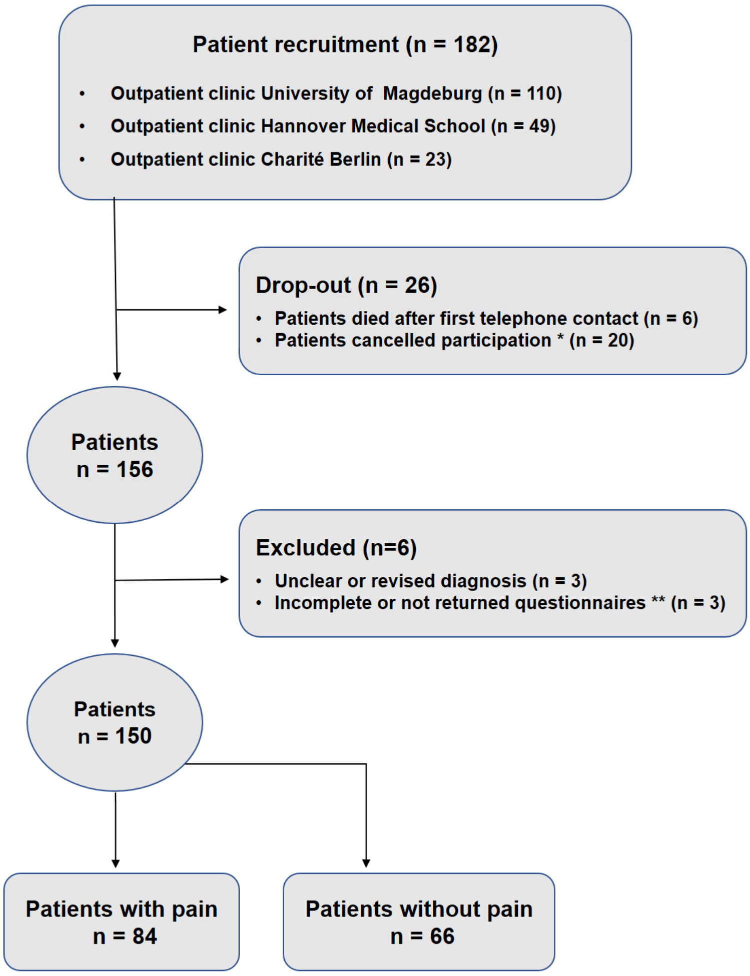

Supplementary Figure S1: Flow diagram of recruitment scheme.
* These patients took part in the telephone interview, but declined to participate in the survey for various reasons, such as fatigue, limited physical capacity and/or the inability to write, especially when there was no one to help answer the questions.
** Either only the telephone interview was conducted, but the questionnaires sent by mail were not answered, or the questionnaires were returned, but only answered incompletely, so that no further conclusions could be drawn from them.
